# Personalised prevention and management of kidney stones: a narrative review on the importance of integrating environmental, social and genetic factors

**DOI:** 10.3389/phrs.2026.1609208

**Published:** 2026-07-10

**Authors:** Aleksandra Leonova, Rosa Sinisi, Stefano Alba, Vincenzo Andracchio, Alberto Piana, Carmela Leonessa, Giuseppina Rose, Vito Summa, Giuseppe Passarino, Paolina Crocco

**Affiliations:** 1 Department of Biology, Ecology and Earth Sciences (DiBEST), University of Calabria, Rende, Italy; 2 Institute of Methodologies for the Environmental Analysis, National Research Council, Tito Scalo, Italy; 3 Romolo Hospital, Rocca di Neto (KR), Italy

**Keywords:** genetic predisposition, kidney stones, personalized medicine, prevention, pro-lithogenic diet, environmental factors

## Abstract

Kidney stone disease (KSD) is a prevalent, multifactorial, and common disorder influenced by environmental and genetic factors, with increasing global incidence and high recurrence rates. Despite advances, understanding the interplay between urinary biochemistry, lifestyle, climate, and genetic predispositions remains essential for effective management. In this review, we evaluate current concepts on KSD pathophysiology, epidemiology, stone composition, and formation mechanisms, alongside recent diagnostic and therapeutic developments. We emphasize integrating genetic, environmental, and clinical data to inform personalized prevention and treatment strategies. These insights support tailored approaches that may improve patient outcomes and reduce recurrence, highlighting the potential of personalized medicine in addressing the complex etiology of KSD.

## Introduction

Blood is filtered through urinary system, where waste products accumulate in the urine. This system includes kidneys, renal pelvis, ureters, urinary bladder, and urethra. Kidneys are the organs responsible for filtration. By removing acids produced by cells, they maintain the balance of water, salts, and minerals in the blood [1]. One common problem in the urinary system is the formation of kidney stones, also called renal calculi, nephrolithiasis, or urolithiasis. Kidney stones are hard deposits, made of minerals and salts that form within the kidneys when there is an imbalance in the concentration of substances present in the urine. Stone formation usually begins when substances such as calcium, oxalate, or uric acid become highly concentrated in the urine. This can occur due to various factors, including insufficient fluid intake, dietary habits, metabolic disorders, urinary tract infections, and medications. Once these substances become highly concentrated, they can crystallize and form small solid particles, which can aggregate, grow, and develop into kidney stones. The size of these stones can vary from small (nanometre-sized) grains to larger (centimetre-sized) solid masses [2]. The stone can be located anywhere between the kidneys and urethra, with symptoms and pains that can vary in relation not only to the size but also to the position of the stones. Typically, stone formation does not cause any symptoms initially. Successively, most common signs and symptoms of the stone disease consist of renal colic (intense cramping pain), pain in the backside (flank pain), blood in the urine (hematuria), urinary tract disease (obstructive uropathy), urinary tract infections, frequent urination or blockage of urine flow, and dilation of the kidney (hydronephrosis). These conditions are often associated with nausea and vomiting, which increase the pain caused by the stone [3]. Therefore, besides the quality of life, treatment and time away from work involve substantial social costs [4]. KSD is the most common chronic disease of the urinary tract, affecting up to 13% of the world’s population [5] and representing a growing burden on healthcare systems worldwide [6, 7]. Moreover, with recurrence rates ranging from 30% to 50% within 5–10 years after the first attack [8], there is an urgent need to find the best means for secondary prevention.

This review brings together recent information on the epidemiology, composition, and types of kidney stones, focusing on biochemical factors and biomineralisation pathways of KSD. Much attention has been paid to risk factors, highlighting the role of climate, trace elements in local environments, lifestyle, and diet. Genetic variability was also analyzed to understand genetic architecture underlying individual predisposition, summarizing recent findings in both rare monogenic conditions of KSD studies and common genes associated with nephrolithiasis, including an overview of Genome-Wide Association Studies (GWAS) in different populations.

Finally, the review discusses how a targeted approach can address different aspects of the disease development based on the comprehensive patient’s risk profiles derived from omics, stone, and genetic data. The future perspective is that with the convergence of environmental, clinical, and genetic data, more personalised and effective strategies can be applied to prevent the global epidemic of kidney stones.

## Epidemiology of kidney stone disease

Kidney stone is a common, complex disease, among the oldest ones identified by medicine [9]. Studying the epidemiology of kidney stones may help to identify high-risk populations, understand the underlying factors contributing to stone formation, and guide preventive strategies to manage and control kidney stone disease. The epidemiology of kidney stones is subject to changing trends over time. Its prevalence and incidence are influenced by several variables, such as age, gender, ethnicity, food heritage, especially in areas of geographical segregation and occupation, but also by multiple environmental factors, including changes in lifestyle and dietary habits, as well as global warming [10–12]. Furthermore, the presence of diseases such as bone fractures, diabetes, hyperparathyroidism, systemic, vascular, and chronic kidney disease is considered a risk factor for kidney stones, and, conversely, urolithiasis is also considered a risk factor for these systemic diseases, perhaps highlighting common risk factors [13–19].

Despite considerable improvements in the development of new therapies, the worldwide prevalence of kidney stones has increased in the last three decades [5]. The prevalence varies across different populations (range 7%–13% globally), with some regions and ethnic groups having higher rates than others. It has been estimated that prevalence rates differ between economically developed and developing countries. This gap, however, could be partly due to higher detection of asymptomatic cases in economically developed countries. In addition, unbalanced dietary habits, such as increased salt and protein intake, together with an increasing prevalence of metabolic syndrome and lack of physical activity, may be one of the causes leading to an increase in the prevalence of urolithiasis in developed countries and, sometimes also in metropolitan areas. Conversely, in developing countries, malnutrition and water deprivation may contribute to the increased prevalence of stone disease [5].

The risk of kidney stone formation progressively increases in the third decade of life and gradually decreases in the seventh [20, 21] with a peak between the ages of 40 and 60 [22]. In particular, it has been estimated that the highest incidence occurs in men aged 20–49 years [11, 23], although the incidence of stone formation is growing among females [24]. Moreover, kidney stone formation is often recurrent during life, and individuals who have had a kidney stone have a higher risk of developing another stone in the future. In particular, stone recurrence rate varies from 30% to 50% within 5–10 years, and 75% within 20 years after the initial stone episode [8].

Due to its high prevalence in working-age adults, kidney stone disease has become a public health issue with a significantly high financial burden on healthcare systems, particularly in populations residing in regions with a hot and dry climate [6, 7].

## Kidney stones: composition and types

Kidney stones differ in chemical and mineralogical composition, size and shape, and position of crystallization within the urinary system. Major inorganic/organic constituents of calculi are calcium phosphate (hydroxyapatite), calcium oxalates (whewellite and weddellite), and urates [25]. Two or more of these components form most of the urinary calculi in various combinations [26]. Among these stone types, calcium stones are certainly the most frequent, accounting for about 70%–80% of all kidney stones.

**FIGURE 1:**
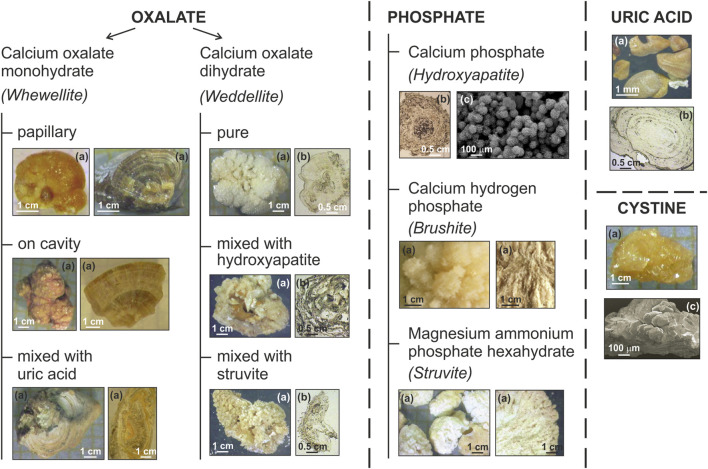
Schematic diagram showing the types and subtypes of urinary stones classified according to [29]. Panels **(a)** and **(b)** display optical microscopy images of bulk samples and thin sections, respectively, while panel **(c)** shows scanning electron microscopy images. Scale bars are provided for all images.

According to several authors [[27, 28] and references therein], 11 main groups of urinary stones may be distinguished (Figure 1) based on mineral composition, presence of trace constituents, site of nucleation, and aetiologic factors [25, 29–32].

From a mineralogical point of view, kidney stones have distinct chemical and structural characteristics that reflect their origin and transformation processes. Table 1 summarizes the principal mineral types identified in kidney calculi, such as calcium, phosphate, urate, cystine, and drug-induced, demonstrating their chemical composition, typical morphology, colour, and internal organization.

**TABLE 1:** Mineralogical composition, morphology, and structural characteristics of main kidney stone types.

Stone type and main mineral	Chemical formula	Morphological characteristics	Colour and appearance	Internal structure	Notes
Calcium oxalate stones: Whewellite – CaOx monohydrate (COM) and weddellite – CaOx dihydrate (COD)	CaC_2_O_4_·H_2_O (COM); CaC_2_O_4_·2H_2_O (COD)	COM: mammillary, locally budding; smooth or rough surfaces COD: well-crystallized bipyramidal aggregates sharing angles/ridges	COM: beige to yellow-brown to brown COD: yellowish to brownish, spiculated	Generally unorganized to poorly organized; occasionally concentric thin layers or radial crystallization	COM and COD commonly coexist in composite stones
Brushite (calcium hydrogen phosphate)	Ca(HPO_4_)·2H_2_O	Finely rough or dappled surface	Whitish to pale beige	Radial crystalline structure	Can transform into the thermodynamically more stable hydroxylapatite through dissolution and reprecipitation
Struvite (magnesium ammonium phosphate)	(NH_4_)Mg(PO_4_)·6H_2_O	Smooth surface	White	Radial growth style	Common in infection-related stones
Urate stones	C_5_H_4_N_4_O_3_ (may occur as anhydrous, dehydrate, urate salts, and ammonium hydrogen urate)	Spherical to ovoid; rough and porous	Orange, brown-orange, beige, or greyish	Porous and poorly organized; thin concentric layers in spherules	May occur in acidic urine; non-opaque in imaging
Cystine	C_6_H_12_N_2_O_4_S_2_	Small grains; rough or smooth surfaces	Creamy to yellowish	Radial or concentric	Associated with cystinuria
Drug-induced stones	Variable (depends on drug)	Irregular; may coat pre-existing calculi	Variable	Variable	Formed from drug crystallization or metabolic interference (e.g., guaifenesin, triamterene, atazanavir, sulfa drugs)

(data from [25, 29, 33–35]).

## Aetiology and formation mechanisms of kidney stones

Urinary supersaturation is the driving force for kidney stone crystallization and consists of a fluid characterized by a content of dissolved substances higher than that normally dissolved under specific conditions. Supersaturation of urine strongly depends on its pH, and the resulting mineralization is determined by both the presence and the specific concentration of stone-forming chemical constituents required for biomineral precipitation. For example, brushite crystals frequently affect patients with hypercalciuria and alkaline urine with pH values higher than 6. Struvite crystallization, instead, occurs in the presence of urine with a pH typically higher than 7, because of the low solubility of phosphate minerals in alkaline fluids. Conversely, uric acid precipitates in the presence of low urine volume and low urine pH (pH < 5); cystine stones form by precipitation and accumulation of cystine not dissolved in urine as a result of an excess of cystinuria in urinary excretions [29].

Multiple modulator molecules also influence crystal formation in the human body. Although they may work differently for everyone, such molecules act as inhibitors or promoters of crystallization. Inhibitors are urine chemicals that prevent crystal formation, from supersaturation to crystal nucleation and growth. Organic (citrate, glycosaminoglycans, glycoproteins), as well as inorganic (pyrophosphates, metallic cations) molecules, play this role in a direct mode, by interacting with crystals, or in an indirect way, by modifying the urinary environment [36]. Conversely, promoters are substances that favor calculi formation by various mechanisms recently overviewed by Bazin et al. [37–39]. Cholesterol, phospho- and glyco-lipids, calcium, sodium, oxalate, cystine, and low urine volume are thought to be the principal chemical promoters. In general, the discrepancy between urinary stone inhibitors and promoters is responsible for stone formation.

According to Worcester [40], one of the most common causes of stone formation is the presence of Randall’s plaques (RPs) [41]. The RPs are suburothelial papillary mineral deposits whose formation is generally associated with low urine volume, low pH, and hypercalciuria. Although the pathogenesis of Randall’s plaque itself is not clearly known and its presence was detected also in most human kidneys without stones, it has been established that RPs may act as nucleating sites for the growth and attachment of idiopathic calcium phosphate kidney stones [42, 43].

In addition to the physicochemical mechanisms and Randall’s plaques, Wang et al. [44] documented the importance of sex hormones, microbiome, and immune response as determining factors of kidney stone formation, although their role is not yet fully understood. For example, emerging data suggest that COM, COD, and urate stones are more common in males, whereas higher percentages of hydroxyapatite and struvite stones can be detected in females [44]. However, how sex differences influence the pathophysiological mechanisms of urinary stone disease is not clear and needs further studies to be explained. Similarly, further and detailed studies are necessary for a comprehensive knowledge of the role and mechanisms of kidney and urinary tract microorganisms, and of immune response in kidney stone formation. Also, it remains unclear the precise mechanism of kidney stone formation via endocytosis. Most researchers agree about the role played by renal tubular cells during the initial phase of kidney stone formation when they may act as the preferred mean for the internalization of CaOx crystals. In addition, it has been suggested that exposure to CaOx crystals induces epithelial cellular injury which is a common predisposing factor to subsequent crystal nucleation and growth [36]. As demonstrated by an *in vitro* study [45], apoptosis at the level of renal tubular cells also may lead to stone formation through cellular demise and postapoptotic necrosis.

## Risk factors

As aforementioned, intrinsic factors such as age, gender, ethnicity, and family background, and extrinsic factors such as climate and environment, lifestyle and dietary habits, occupation, and education, are considered risk factors for kidney stone formation. In the following lines, social habits, environmental factors, genetic basis, and comorbidities concerning the risk of developing kidney stones will be analyzed.

### Environmental and lifestyle factors

It is well known that urolithiasis is directly linked to environmental factors that characterize the site where people live. Among these, climate, water, and soil quality are the most important [46–48]. According to Liu et al. [49] and several other authors [50–56], a geographic variability of stone disease, associated with a positive correlation between high temperature and the development of kidney stones, exists. Individuals living in countries or regions falling in tropical and subtropical areas have a higher probability (from 5% to 10%) of developing urolithiasis than those in temperate and cold zones (from 1% to 5%). The cause of such a geographic dependency is the hot, dry conditions that enhance body water evaporation and consequently favor urine concentration. Additionally, it is worth noting that, under the same climate conditions, the incidence of urolithiasis is higher in summer and autumn than in spring and winter [49]. This may also be related to higher sunlight exposure, which stimulates vitamin D synthesis and may increase intestinal calcium absorption, thereby contributing, in predisposed individuals, to urinary supersaturation with respect to calcium-bearing oxalates and phosphates. However, the formation of kidney stones, especially uric acid ones, may occur in low-temperature environments as well. Giannossi et al. [57] documented that in cold zones (i.e., areas with low mean yearly temperatures) one of the determining factors of stone disease seems to be the poor beverage intake, which is responsible for a low urine volume and thus for the urine oversaturation that may lead to salts and crystals precipitation.

Drinking water and foods are the main carriers of the most important organic and inorganic nutrients (protein, vitamins, and minerals inherited from the geoenvironmental matrices) [58] to the human body. In the last decades, the role of major and trace elements in urolithiasis has been widely investigated. One of the most studied natural factors is the water hardness, which mirrors the presence and amount of Ca^2+^ and Mg^2+^ dissolved ions in water. Although previous studies have documented contrasting results about the impact of Ca- and Mg-rich drinking water on urolithiasis [59], the kidney stones composition attests that the most relevant element for urinary lithogenesis is certainly calcium. Its presence may influence the distribution of other elements occurring in the kidney system and promote crystallization processes [[60], and references therein]. However, calcium input to humans is linked to the water as much as the food intake. In the past, diets with very low calcium intake, including marked restriction of milk and dairy products, were considered a possible strategy to reduce stone recurrence [61, 62]. However, current evidence indicates that dietary calcium should not be indiscriminately restricted in calcium oxalate stone formers. When consumed with meals, calcium binds oxalate in the intestinal lumen, reducing intestinal oxalate absorption and urinary oxalate excretion [63–65]. Accordingly, a normal-calcium diet combined with reduced salt and animal protein intake has been shown to be more effective than a low-calcium diet in preventing recurrent calcium oxalate stones in hypercalciuric patients [66]. Excessive sodium intake should also be limited, because it increases urinary calcium excretion and may therefore enhance lithogenic risk [67]. An excess of dietary oxalate remains a relevant predisposing factor, especially when associated with reduced fluid intake. Oxalate-rich foods include, among others, spinach, rhubarb, nuts, chocolate, tea, and some carbonated beverages [68, 69]. As for calcium, the trace elements (i.e. heavy metals) present in water and foods may act as triggering factors for kidney stones formation. Several authors [7, 57, 70, 71] demonstrated that such elements preferentially concentrate in the nucleus or specific accretion layers of urinary crystals, thus representing reliable markers of environmental risks of urolithiasis and a valid tool for biomonitoring aims [60, 72–76].

In this context, dietary habits are also considered as behavioral risk factors inducing stone growth [69, 77]. Studies have shown that a diet rich in meat leads more frequently to the development of stones. Due to excessive protein consumption, urine becomes more acidic, and the elimination rate of oxalates, calcium, and uric acid increases, while the rate of citrates (substances that prevent the precipitation of these salts) decreases [78, 79].

A role in stone formation is also played by the work environment and mainly by its microclimatic conditions. The factors that influence the work environment are air temperature, relative humidity in the room, ventilation, intensity of physical effort (energy expenditure), and clothing (thermal impedance of clothing), which may induce a series of graded biological responses in humans [55].

The prevalence rate of nephrolithiasis also varies depending on the level of education and profession. The prevalence is high among people with no schooling or with a low level [80]. As to professions, the most at risk are those that involve sitting for long periods, in sedentary positions, or overheated environments, the last for the same reasons as for climatic factors [81–83].

### Genetic basis of kidney stone formation

Several studies have shown that people with a family history of kidney stones have a higher risk of developing kidney stones than patients without a family history, suggesting a strong genetic component to kidney stone formation [84, 85]. Twin studies have shown that identical twins have a higher risk of developing kidney stones compared to dizygotic twins [77, 86], suggesting a heritability of kidney stones of around 30%–60%. Genetic factors, while important, often interact in complex ways with environmental and lifestyle factors to determine an individual’s risk for developing kidney stones, making prevention difficult. In recent decades, important progress has been made in understanding the genetic basis of kidney stones. For instance, there are rare genetic disorders, such as primary hyperoxaluria and Dent’s disease, characterized by a strong genetic basis for kidney stone formation. These conditions are caused by mutations in specific genes that affect the management of minerals in the body, leading to kidney stone formation. Table 2 shows the main monogenic urolithiasis disorders related to the type of stone, the genes involved, the type of inheritance, and the related phenotype. In a recent review of monogenic features of urolithiasis, Koo and colleagues [103] reported some evidence obtained from high-throughput sequencing technologies. A prospective study found that 11.4% of adults and 20.8% of paediatric urolithiasis cases were due to a single gene mutation [109]. The monogenic causative mutations were detected in 46.7% of the analyzed genes, with a molecular diagnosis in 16.8% of the patients [110]. In addition, a whole-exome sequencing carried out in 51 families in which at least one kidney stone occurred before the age of 25 years, evidenced a monogenic causative mutation in 29.4% of the families suggesting that young age at diagnosis of kidney stones, the presence of multiple affected family members, and consanguinity as factors associated with a higher rate of monogenic mutations [111].

**TABLE 2:** Monogenic disorders of urolithiasis.

Disorder	Type of stones	Gene	Inheritance	Phenotype	References
Autosomal dominant hypocalcemia (ADH) with hypercalciuria	Calcium-containing stones	Type 1 – activating mutations of the *CASR* Type 2 - *GNA11*	AD	Mild to moderate hypocalcemia, hyperphosphatemia, hypercalciuria, low but detectable level of parathyroid hormone (PTH), hypocalcemic symptoms	[87]
Familial hypocalciuric hypercalcemia	Calcium-containing stones	Inactivating mutations of the *CASR*	AD	Mild hypercalcemia, hypermagnesemia, hypocalciuria, and hypophosphatemia, normal or increased levels of PTH	[88, 89]
Idiopathic hypercalciuria	Calcium-containing stones	*ADCY10* and *VDR*	AD	Normocalcemia, normal level of PTH, often low bone mineral density	[90]
Bartter syndrome Type 1	Calcium-containing stones	*SLC12A1*	AR	Polyuria, hypochloremia, alkalosis, hypokalemia, hypercalciuria followed by nephrocalcinosis	[91]
Bartter syndrome Type 2	Calcium-containing stones	*KCNJ1*	AR	Polyhydramnios, intrauterine growth retardation, prematurity, developmental delay, polyuria, hypercalciuria followed by nephrocalcinosis	[91, 92]
Bartter syndrome Type 3	Calcium-containing stones	*CLCNKB*	AR	Failure to thrive, low chloremia and severe hypokalemic alkalosis	[93]
Bartter syndrome Type 4	Calcium-containing stones	*BSND* or simultaneous mutations in *CLCNKB* and *CLCNKA*	AR	Sensorineural hearing loss, polyuria, hypochloremia, alkalosis, hypokalemia	[91, 94]
Bartter syndrome Type 5	Calcium-containing stones	*MAGED2*	XLR	Polyuria, hypochloremia, alkalosis, hypokalemia	[91]
Dent disease (DD)	Calcium-containing stones	*Type 1 - CLCN5* *Type 2 - OCRL*	XLR	Low-molecular-weight proteinuria, hypercalciuria, and at least one of the following manifestations, nephrocalcinosis, nephrolithiasis, hematuria, hypophosphatemia, or renal insufficiency Type 1%–65% of all cases of DD Type 2%–10%–15% of all cases of DD	[95]
Hereditary hypophosphatemic rickets with hypercalciuria	Calciumcontaining stones	*SLC34A3*	AR	Renal phosphate wasting leading to hypophosphatemia, rickets, limb deformities, muscle weakness, bone pain, renal calcification and hypercalciuria caused by elevated serum 1,25-dihydroxyvitamin D level and calcium absorption in intestine	[96, 97]
Familial hypomagnesemia with hypercalciuria and nephrocalcinosis	Calcium-containing stones	*CLDN16* and *CLDN19*	AR	Nephrocalcinosis due to calcium and magnesium wasting. In the case of *CLDN19* mutation severe ocular abnormalities	[98, 99]
dRTA (distal renal tubular acidosis)	Calcium-containing stones	*SLC4A1* *ATP6V1B1* *ATP6V0A4, FOXI1, WDR72*	AR (AR or AD in the case of *SLC4A1*)	Hyperchloraemic metabolic acidosis, hypercalciuria, hypocitraturia, nephrocalcinosis, polyuria, sensorineural deafness, growth delay	[100, 101]
Primary hyperoxaluria	Calcium oxalate stone	Type 1 – *AGXT* Type 2 – *GRHPR* Type 3 - *HOGA1*	AR	Excessive production of oxalate, urinary oxalate excretion, increased risk of nephrocalcinosis and calcium oxalate stones formation risk of oxalosis	[90]
Infantile hypercalcemia	Calcium-containing stones	*SLC34A1* and *CYP24A1*	AR	Nephrocalcinosis, hypercalcemia, hypercalciuria, suppressed parathormone, increased or normal serum 1,25 (OH)2-vitamin D3, renal phosphate wasting	[102]
Cystinuria	Cystine stones	Type A - *SLC3A1* Type B - *SLC7A9*	Type A – AR Type B – AR or AD	Increased risk of cystinuria and nephrocalcinosis	[90, 99]
HPRT1 disorders. Three phenotypes: Lesch-Nyhan disease (LND), HPRT1-related neurologic dysfunction (HND), HPRT1-related hyperuricemia (HRH)	Uric acid stones	*HPRT1*	XLR	Hyperuricemia, nephrolithiasis, and/or gouty arthritis, hyperuricosuria and neurological and behavioral deficit	[103, 104]
Renal hypouricemia (RHUC)	Uric acid stones	*SLC22A12* and *SLC2A9*	AR	Urolithiasis, nephrolithiasis, kidney injury, hypouricemia, increased fractional excretion of uric acid	[105, 106]
Hereditary xanthinuria	Xanthine stones	Type 1 -*XDH* Type 2 - *MOCOS*	AR	High amount of xanthine in the urine, decreased level of uric acid in serum and urine	[107]
Adenine phosphoribosyltransferase (APRT) deficiency	2,8-dihydroxyadenine stones	*APRT*	AR	Kidney stones formation, chronic kidney disease, crystal nephropathy	[108]

* AD, autosomal dominant.

** AR, autosomal recessive.

*** XLR, X-Linked Recessive.

Although the exact number of genes involved is not yet defined, one study demonstrated at least 15% of kidney stone cases were due to causative mutations in 14 genes [109]. On the other hand, in most patients with kidney stones, even with a strong family history, a single genetic cause for the increased risk of stone formation is not found [55]. Besides, family analysis revealed that the inheritance pattern of calcium stones is not mendelian, but rather complex and affected by multiple genes [112, 113]. These findings suggest that nephrolithiasis has multifactorial causes for most patients involving the interaction of genetic and environmental factors.

Based on biological functions, case-control studies have examined the role of biologically relevant candidate genes in nephrolithiasis development, identifying associations with genes such as *SLC26A6*, *TRPV5*, *TRPV6*, *VDR*, *HIPK2*, *OPN*, *MGP*, and *PLAU* [[90], and references therein]. Table 3 reports the main renal phenotypes related to these candidate genes.

**TABLE 3:** Candidate genes related to kidney stones phenotypes.

Candidate gene	Gene product	Renal phenotype
*SLC26A6*	Solute carrier family 26, member 6	An anion exchanger (chloride, formate, bicarbonate, sulfate, hydroxyl and oxalate) that may affect hyperoxaluria [114]
*TRPV5*	Transient receptor potential cation channel subfamily V member	A calcium channel that is expressed in distal convoluted tubule and connecting tubule and regulates the entry of calcium into the cell. It is associated with recurrent kidney stones and urinary calcium excretion, familial stone disease, or stone multiplicity and is involved in the process of vitamin D-responsive active Ca2+ reabsorption in the kidney [115, 116]
*TRPV6*	Transient receptor potential vanilloid subfamily member 6	A calcium channel that is mainly expressed in the intestine, with additional expression in the kidney. Activating variants of this channel are linked to increased Ca^2+^ transport (absorptive hypercalciuria) and risk of renal stone [88, 117, 118]
*VDR*	Vitamin D Receptor	A ligand-activated transcription factor which is activated by 1,25-Dihydroxyvitamin D (1,25(OH)2D3). In the kidney, it is expressed in proximal and distal tubular epithelial cells, podocytes, and collecting duct epithelial cells, and is strictly associated with kidney stone formation [119, 120]
*HIPK2*	Homeodomain-interacting protein kinase 2	Acts as a signaling effector and transcriptional coactivator/corepressor. Its expression is increased in kidney epithelial cells in CKD. Some variants are strictly associated with KSD in males [121, 122]
*OPN*/*SPP1*	Osteopontin	Involved in inflammation, cell survival, and crystalline interaction. It promotes the deposition and attachment of crystals during the initial phase of calcium oxalate stone formation and is highly expressed in the distal tubule cells of stone-forming rats [99]
*MGP*	matrix Gla protein	A vitamin K-dependent inhibitor of vascular calcification that suppresses the calcium oxalate monohydrate formation and nucleation of hydroxyapatite [90, 123]
*PLAU*	Urokinase-type plasminogen activator	Reported to be associated with calcium nephrolithiasis in Taiwan and Turkey, it may also protect against stone formation through matrix protein degradation [124, 125]

Additionally, various GWAS have explored the genetic variability of nephrolithiasis, identifying different genes and variants across diverse populations. Table 4 summarizes the main findings, detailing the analyzed populations, associated genes, and identified polymorphisms. As shown in the table, associated genes vary across studies. These differences likely reflect the unique genetic architecture of each population and the influence of diverse environmental factors on this complex phenotype. Moreover, the identification of different genes may also stem from the various approaches used: some studies performed subgroup GWAS based on stone compositions (e.g., calcium stones), uncovering unique genetic risk factors specific to certain populations.

**TABLE 4:** List of kidney stones Genome-Wide Association Studies reporting information about population, genes, polymorphisms, and references.

Population	Genes	rs	References
Iceland	*CLDN14* *	rs219779 rs219780	[126]
Iceland	*UMOD* * (protective in the case of KSD but risk variant in CKD)	rs4293393	[127]
Japan	*RGS14-SLC34A1-PFN3-F12* * (upstream of the *SLC34A1* gene) *INMT-FAM188B-AQP1* * (upstream of the *AQP1* gene) *DGKH* *	rs11746443 rs1000597 rs4142110	[128]
Iceland	*CLDN14* *ALPL* *SLC34A1* *CASR* (suggestive association)	rs199565725 rs1256328 rs12654812 rs7627468	[129]
Iceland	*POU2AF1* (suggestive association)* *WDR72* *	rs12417556 rs551225	[130]
The UK and Japan	*ALPL* *GCKR* *DGKD* * *ABCG2* * *SLC34A1* *KCNK5* *SLC22A2* * *HIBADH* * *AQP1* *POU2AF* *DGKH* *WDR72* *UMOD* *SCNN1B* * *BCAS* *SOX9* * *GIPC1* *CYP24A1* *CLDN14* *BCR* *	rs10917002 rs780093 rs13003198 rs1481012 rs56235845 rs1155347 rs77648599 rs12539707 rs12666466 rs4529910 rs1037271 rs578595 rs77924615 rs889299 rs1010269 rs4793434 rs3760702 rs17216707 rs12626330 rs13054904	[131]
Japan	*ALPL* *GCKR* * *MRPL33* * *RGS14* *KCNK5* * *TFAP2B* * *EPB41L2* * *INMT-FAM188B* *DGKH* *PDILT* * *FTO* * *BCAS3* * *LOC645722* * *PKN1* * *BCAS1* * *BCAS1* * *CLDN14*	rs6667242 rs1260326 rs13006480 rs11746443 rs1544935 rs3798519 rs6928986 rs6975977 rs7328064 rs35747824 rs7206790 rs2079742 rs2286526 rs74956940 rs13041834 rs6123359 rs7277076	[132]
Japan	*STIM1* * *DGKH* *PDILT* *BCAS3* *PKN1* *GCKR* *BCAS1,CYP24A1* *ABCG2* *RGS14* *FAM188B*	rs12290747 rs7328064 rs62034975 rs2079742 rs60984983 rs10549495 rs35194449 rs4148155 rs11741640 rs73301967	[133]
Europe (Finland, the UK), East Asia (Japan)	*ALPL* *RNU5F-1* * *ERBB4* * *DGKD* *** *GCKR* *ZFP36L2* * *CASR* * *UGT8* * *SHROOM3* * *ABCG2* *RGS14* *EPB41L2* *SLC22A2* *** *HLA-DQA1* *KCNK5* *TFAP2B* *HCRTR2* * *HIBADH* *** *INMT-MINDY4* *VPS13B* * *NIPAL2* * *FAM35A* * *DGKH* *CLDN10* * *MIPOL1* * *WDR72* *** *PDILT* *ZFPM1* * *LINC00670* * *BCAS3* *LINC02003* * *GIPC1* *BCAS1* *CLDN14* *RSPH14* *	rs13353032 rs884127 rs147025686 rs838717 rs1260326 rs1430083 rs10602692 rs4834412 rs11723275 rs2231142 rs11748297 rs35250412 rs141163734 rs9271375 rs62398607 rs3798519 rs79986767 rs5883088 rs6975977 rs557293205 rs118079955 rs6586064 rs9594689 rs9525016 rs1950526 rs578595 rs77924615 rs55637647 rs2726550 rs9895661 rs56008432 rs2241358 rs6123359 rs7277076 rs13054904	[134]
Europe (Finland, the UK)	*ANXA9* * *PACERR* * *SLC41A1* * *SLC30A10* * *PRKAR2A* * *DOCK3* * *POC1A* * *ADRA2C* * *SHROOM3* *** *UGT8* *** *ISL1* * *PRRT1* * *VEGFA* * *TFAP2B* *** *ASCC3* * *TRPV5* * *TMEM252* * *AOPEP* * *TCEB1P3* * *RNLS* * *CLDN10* *** *SLC12A1* * *PDE8A* * *ZFPM1* *** *MAPT* * *STAP2* * *GIPR* * *ZNF468* * *ALPL* *DGKD* *CASR* *ABCG2* *RGS14* *KCNK5* *SLC22A2* *HIBADH* *AQP1* *DGKH* *PDILT* *BCAS3* *LINC00511* *CYP24A1* *RSPH14*	rs267733 rs113831804 rs823121 rs884127 rs200495345 rs191107165 rs138789058 rs440318 rs28454965 rs71606723 rs55672774 rs3134962 rs729761 rs2206271 rs1039031 rs4252512 rs12376362 rs150891531 rs17486892 rs11202736 rs57719175 rs34819316 rs10852147 rs55637647 rs242559 rs58169740 rs1800437 rs7259073 rs10917002 rs13003198 rs7627468 rs1481012 rs56235845 rs1155347 rs77648599 rs12539707 rs1000597 rs1037271 rs77924615 rs1010269 rs4793434 rs17216707 rs13054904	[135]
The UK	*ALPL* *ALPL* *DGKD* *SLC34A1* *KCNK5* *SLC2A12* * *SLC22A2* *HIBADH* *TRPV5* *DGKH* *WDR72* *SLC28A1* * *UMOD* *CYP24A1* *CLDN14* *BCR*	rs77362499 rs1256332 rs838717 rs56235845 rs1155347 rs969282 rs78693187 rs7790498 rs4252512 rs1182959 rs578595 rs12439802 rs77924615 rs6127099 rs2776288 rs13054904	[136]
Taiwan**	*NFACT1* * *PCDH15* * *DGKH* *ABCG2* *** *PDILT* *BCAS3* *HDAC4* * *RN7SKP27* * *AP003068.2* *	rs71359461 rs1935910 rs9533022 rs141471965 rs79746097 rs9895661 rs6543514 rs140022940 rs1195967	[137]
the Southeastern USA (electronic health record-based datasets)	*UMOD* *UMOD* *UMOD* *UMOD* *UMOD* *UMOD* *UMOD* *UMOD* *UMOD* *UMOD* *UMOD* MIR4455; HELT**** MIR4455; HELT**** OVCH2; OR5P2**** LINC01031; LINC01724**** LINC01031; LINC01724**** LINC01031; LINC01724**** LINC01031; LINC01724**** LINC01031; LINC01724**** NXPH1**** NXPH1**** FAM86C2P; UNC93B1**** FAM86C2P; UNC93B1**** FAM86C2P; UNC93B1**** MIR8054; LUZP2****	rs28544423 rs9928003 rs13335818 rs34882080 rs35650857 rs12934320 rs34356953 rs71149135 rs60136849 rs111285796 rs56193428 rs56193428 rs72706967 rs369841339 rs34398946 rs35337461 rs12748379 rs71642955 rs35647468 rs79970906 rs4725104 rs148417243 rs144507654 rs141950436 rs72880913	[138]
​	FAM86C2P; UNC93B1**** FAM86C2P; UNC93B1**** DUSP26; LINC01288**** LINC01288**** OXTR; RAD18****	rs12274909 rs12273415 rs186944649 JHU_8.34686048 rs143825102	​

* novel association.

** only calcium stones.

*** also identified as a novel association in the study but is present in earlier GWAS, also as novel.

****subgroup GWASs, stratified by subtypes of kidney stone composition.

Taken together, these results indicate that genes involved in kidney stone predisposition are primarily implicated in calcium and phosphate regulation, metabolic traits, and oxidative stress.

## Discussion

**FIGURE 2:**
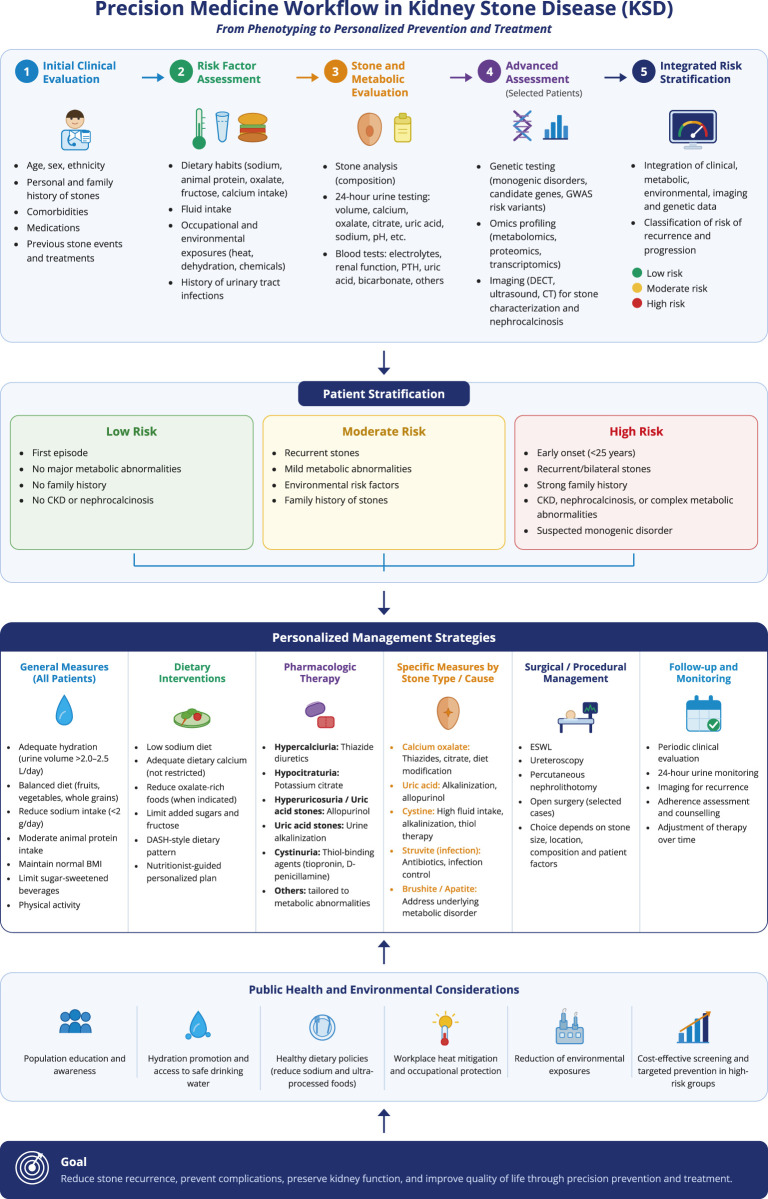
Schematic representation of a personalized approach to the prevention, diagnosis, and management of kidney stone disease integrating clinical phenotype, dietary and environmental exposures, metabolic assessment, stone composition analysis, imaging, and genetic evaluation. The framework highlights a stepwise patient stratification process aimed at identifying individualized preventive and therapeutic strategies according to stone phenotype, metabolic abnormalities, and inherited susceptibility. Personalized interventions may include nutritional modifications, hydration strategies, pharmacological treatments, and targeted management of monogenic disorders, with the overall goal of reducing recurrence risk and improving long-term clinical outcomes.

The concepts summarized in the previous sections highlight the complexity of kidney stone disease (KSD) pathophysiology and support the view that KSD should increasingly be considered a heterogeneous and multifactorial disorder requiring individualized diagnostic and therapeutic strategies rather than a uniform management approach (Figure 2).

From a public health perspective, population-based prevention strategies, including hydration campaigns, reduction of dietary sodium and ultra-processed food consumption, workplace heat mitigation measures, and improved access to nutritional counseling, may substantially reduce the burden of KSD, particularly in high-risk geographic regions affected by climate change. Successful prevention of kidney stones requires the analysis of different aspects that contribute to stone formation to identify personalized strategies based on individual characteristics and risk factors, such as those shown in this review. Preventing primary KSD through dietary changes, for instance, is a cost-effective public health measure with a significant societal impact. In addition, nowadays, several efficient treatments exist for kidney stone disease. The most common approaches involve surgical removal procedures such as extracorporeal shock wave lithotripsy, ureteroscopy, percutaneous nephrolithotomy, and/or open surgery. The choice of treatment mainly depends on the stone’s size, composition, location, urinary tract anatomy, and the patient’s morphology. Medical management of urolithiasis often involves drugs or invasive surgical procedures that may cause side effects or complications, such as hemorrhage, hypertension, tubular necrosis, subsequent kidney fibrosis, renal failure, steinstrasse (small stones blocking the ureter), pancreatitis, infections, and persistent residual stones, which can act as a nidus for new stone formation. Stone recurrence after removal continues to be a significant challenge in the surgical management of kidney stone disease. A study carried out in Iceland, has revealed that the recurrence rate among children after stone removal varies from 26% at 5 years post-surgery to 46% at 20 years post-surgery [139, 140]. Therefore, research into alternative treatment and prevention methods remains an avenue worth exploring to prevent recurrence. Alternative methods, particularly those involving medicinal and aromatic plants (MAP), are prevalent worldwide, especially in developing countries. Ethnobotanical studies conducted in Morocco have revealed the potential of certain plants for treating lithiasis, preventing recurrence, and alleviating renal colic. *In vitro* studies have shown that plant extracts can inhibit stone formation at various stages of crystallization (nucleation, growth, aggregation). Examples of these plants include *Rotula aquatica*, *Holarrhena antidysenterica*, *Origanum vulgare*, *Ammi visnaga*, *Phyllanthus niruri*, *Herniaria hirsuta*, *Apium Graveolens,* and others, which have shown activity against calcium oxalate crystals. Other plants, like *Commiphora wightii* and *Citrus medica*, have been found to inhibit struvite crystal growth. Moreover, essential oils from *Mentha pulegium* and *Eucalyptus camaldulensis* have shown significant activity against bacteria responsible for infection-induced lithiasis. *In vitro* studies using cell culture models and animal studies also demonstrate the preventive effects of these plants on cell damage and stone formation [141]. However, further research is required to test and validate the efficacy of these traditional resources.

Precise identification of the primary cause of nephrolithiasis is crucial for determining the suitable treatment approach [142, 143]. Although common strategies like ensuring hydration can help to reduce stone formation in all patients, personalized therapies can be more appropriate for specific types of stones [142]. In this context, precision medicine may provide an actionable framework integrating clinical phenotype, metabolic abnormalities, dietary habits, environmental exposures, stone composition, and genetic susceptibility to improve prevention and treatment outcomes [142]. A stepwise patient stratification model may support clinical decision-making. Initial evaluation should include detailed phenotypic characterization, including age at onset, recurrence rate, family history, comorbidities, occupational heat exposure, dietary patterns, and previous urinary tract infections. Stone composition analysis and metabolic assessment through 24-h urine testing remain central for identifying lithogenic risk factors such as hypercalciuria, hyperoxaluria, hypocitraturia, and hyperuricosuria. In the case of non-calcium stone formers, the understanding of the composition of stones is essential, as it gives crucial diagnostic insights. For example, discovering a stone composed entirely of cystine or even the traces of cystine confirms cystinuria, an inherited autosomal recessive disorder. Additionally, detecting a mixed stone of struvite and calcium oxalate strongly indicates the presence of a metabolic disorder [144]. While the presence of apatite as a main component might suggest conditions like renal tubular acidosis or primary hyperparathyroidism, however, this is not always certain. The existence of brushite indicates a more aggressive form of stone disease that is less responsive to preventive measures and raises the risk of CKD [145].

In most cases, calcium stones have a mixed composition reflecting the underlying pathogenetic mechanisms and/or simultaneous lithogenic processes involved in kidney stone formation. The calcium content of a stone can often be inferred from a plain abdominal X-ray of radiopaque stones, while dual-energy CT appears to be promising as a useful diagnostic tool for detecting uric acid stones [2, 146].

Ferraro et al. [143] provided a comprehensive review of clinical manifestations suggesting inherited disorders, such as hypophosphatemia, low-grade proteinuria, and renal hyperechogenicity. Some of these patients have a monogenic cause, which means they could benefit from genetic screening. Although today’s costs are too high for most individuals, academic funding and clinical research are anticipated to improve access to genetic testing by demonstrating its cost-benefit [147].

In extreme cases, failing to diagnose a monogenic stone-forming condition like primary hyperoxaluria or adenine phosphoribosyltransferase (APRT) deficiency can have serious consequences. For instance, overlooking a diagnosis of primary hyperoxaluria type 1 may result in the recurrence of the disease and oxalosis in a transplanted kidney [143], and the failure to consider the option of curative liver transplantation. In some cases, undiagnosed APRT deficiency can result in end-stage renal disease (ESRD) and is linked to the recurrence of stone disease in a renal transplant, which may cause the loss of graft function if it remains unaddressed [148].

Diagnosing such cases depends on a combination of different methods, such as stone investigation, urine biochemistry, and genetic screening. In the case of stone formers, precision medicine approaches are not only genetics-based; they also employ a wide range of phenotypic information [142]. Genetic screening may have potential in improving the clinical management of kidney stone disease. Despite the well-established contribution of genetics to stone formation, its integration into routine clinical care remains limited. It is important to note that many rare genetic causes of stones and other renal conditions have metabolic signs, which should be detected and diagnosed through clinical observations and systematic metabolic testing. Extensive lists of recognized monogenic causes of stones have been issued [88, 149].

In selected high-risk individuals, including patients with early-onset disease, nephrocalcinosis, recurrent bilateral stones, chronic kidney disease, or strong familial aggregation, genetic testing may help identify monogenic disorders and guide targeted management strategies [88]. There is a pressing need to translate these findings into clinical practice by evaluating genetic variants in real-world patient populations and correlating them with clinical phenotypes, including disease severity and stone composition. Integrating kidney-specific omics data has further highlighted the role of ion homeostasis, particularly calcium and magnesium, in stone formation, suggesting novel therapeutic targets. Additionally, stronger genetic correlations between kidney stone disease and genitourinary or digestive diseases underscore the multifaceted nature of stone pathogenesis. Therefore, comprehensive genetic screening could inform personalized treatment strategies and may help optimize existing treatment algorithms.

Within this framework, a personalized approach to the prevention, diagnosis, and management of kidney stone disease can be tailored according to the underlying pathogenic mechanisms and stone phenotype (Figure 2). For example, urine alkalinization may be particularly effective in uric acid stone formers, whereas thiazide diuretics and dietary sodium restriction may benefit patients with hypercalciuria. Similarly, potassium citrate supplementation may be indicated in hypocitraturic patients, while individuals with cystinuria often require intensive hydration and thiol-based therapies. Environmental and public health factors should also be integrated into individualized prevention strategies, particularly in populations exposed to chronic dehydration, high ambient temperatures, or limited access to healthy dietary resources. However, implementing personalized prevention strategies in routine clinical practice remains challenging because of poor long-term adherence to dietary modifications, difficulties in accurately assessing nutritional habits, variability in 24-h urine collections, and limited access to specialized multidisciplinary care. In addition, despite promising advances in genomics and omics technologies, important barriers still limit the implementation of precision medicine in clinical practice, including costs, limited accessibility to genetic testing, variability in patient adherence, and the lack of standardized clinical algorithms. Nevertheless, integrating phenotypic, metabolic, environmental, and genetic information may contribute to more accurate risk stratification, earlier diagnosis, reduced recurrence, and optimization of long-term management in patients with KSD.

### Conclusion

In conclusion, the multifactorial aetiology of kidney stone formation highlights the need for diverse and integrative approaches to comprehend its aetiology and apply the knowledge within the precision medicine framework. The decisive impact of metabolic-targeted dietary patterns and nutritional recipes, acting in synergy with epigenetic modulations and environmental determinants, constitutes a fundamental pillar of kidney stone formation. Acknowledging this multifactorial interplay is essential for transitioning toward an actionable precision medicine framework. At the same time, uncovering the genetic underpinnings of the disorder may enable early identification of individuals at elevated risk, paving the way for novel diagnostic tools, targeted therapies, and customized prophylactic measures for those prone to recurrent urolithiasis.
